# Learning from the implementation of a quality improvement intervention in Australian general practice: a qualitative analysis of participants views of a CVD preventive care project

**DOI:** 10.1186/s12875-022-01692-0

**Published:** 2022-04-14

**Authors:** C. M. Hespe, E. Brown, L. Rychetnik

**Affiliations:** 1 Department of General Practice, University of Notre Dame Australia, Sydney, NSW Australia; 2 The Australian Health Prevention Partnership, Sax Institute, Sydney, NSW Australia; 3 School of Public Health, Faculty of Medicine and Health, The University of Sydney, Sydney, NSW Australia

**Keywords:** Primary care, Implementation, Quality improvement, Cardiovascular disease, Cardiovascular disease prevention, Preventive care, General practice, Quality improvement collaboration, Primary health network, PHN

## Abstract

**Background:**

Quality improvement collaborative projects aim to reduce gaps in clinical care provided in the healthcare system. This study evaluated the experience of key participants from a Quality Improvement Program (QPulse) that focussed on cardiovascular disease assessment and management. The study goal was to identify critical barriers and factors enabling the implementation of a quality improvement framework in Australian general practice.

**Methods:**

This qualitative study examined in-depth semi-structured interviews with nineteen purposively-selected participants of the QPulse project. Interviewees were from General Practices and the local supporting organisation, a Primary Health Network. Interviews were analysed thematically using the Complex Systems Improvement framework, focusing on five domains: strategy, culture, structure, workforce and technology.

**Results:**

Despite reported engagement with QPulse objectives to improve cardiovascular preventive care, implementation barriers associated with this program were considerable for all interviewees. Adoption of the quality improvement process was reliant on designated leadership, aligned practice culture, organised systems for clear communication, tailored education and utilisation of clinical audit and review processes. Rather than practice size and location, practice culture and governance alignment to quality improvement predicted successful implementation. Financial incentives for both general practice and the Primary Health Network were also identified as prerequisites for systematised quality improvement projects in the future, along with individualised support and education for each general practice. Technology was both an enabler and a barrier, and the Primary Health Network was seen as key to assisting the successful utilisation of the available tools.

**Conclusions:**

Implementation of Quality Improvement programs remains a potential tool for achieving better health outcomes in General Practice. However, enablers such as financial incentives, individualised education and support provided via a supporting organisation, and IT tools and support are crucial if the full potential of Quality Improvement programs are to be realised in the Australian healthcare setting.

**Trial registration:**

ACTRN12615000108516, UTN U1111-1163–7995.

**Supplementary Information:**

The online version contains supplementary material available at 10.1186/s12875-022-01692-0.

## Introduction

Cardiovascular diseases (CVD) are the single leading cause of death in Australia and most developed countries, despite significant declines in morbidity and mortality over the last 40 years [1]. In 2015, CVD was responsible for 29% of deaths and over 1.1 million hospital admissions [2, 3]. Importantly, CVD burden can be reduced through risk-factor modification [4]. Around two-thirds of Australians have three or more modifiable risk factors such as tobacco smoking, high blood pressure, high cholesterol, physical inactivity, poor nutrition, or overweight/obesity [3, 5, 6]. General Practitioners (GPs) play a significant role in mitigating CVD morbidity and mortality, seeing over 85% of the population and conducting over 20 million patient consultations annually [7]. However, previous studies have shown sub-optimal measurement and management of CVD risk in Australian and International primary care settings [8–13]. The 2021 Australian Institute of Health and Welfare data from more than 5700 Australian general practices recorded only 48.5% of patients with enough data to measure cardiac risk [14].

Quality Improvement (QI) initiatives in primary care have the potential to improve uptake of evidence-based practices [15]. QI is a multi-dimensional concept, which can be defined as having a systematic approach to making changes that will lead to better patient outcomes (health), better system performance (care) and better professional development (learning) [16]. Bataldan et al. postulate that defining QI in this way allows people to have a measurable approach to the concept of improving healthcare [16]. There are several ways to implement QI initiatives intentionally, and one such method is establishing a Quality Improvement Collaborative (QIC) [17]. QICs actively bring together practitioners from different organisations to meet and learn about a specific aspect of health service quality and share experiences about making changes to improve measurable outputs in their local settings. There has been mixed evidence of success implementing QICs in health care [15, 18, 19]. However, a systematic review of 64 QIC programs in 2018 reported significant improvements in 83% of targeted clinical processes and patient outcomes [20].

There have been several programs aimed at improving the adoption of guidelines using the QIC framework in the Australian healthcare setting [21, 22]. However, there is a gap in understanding the barriers or enablers for implementation.

This paper presents a qualitative analysis of interviews with participants of a QIC project, QPulse, focused on enhancing the implementation of the Australian National Vascular Disease Prevention Alliance Guidelines^1^ to manage absolute cardiovascular disease risk [23]. QPulse was a collaboration between the Central and Eastern Sydney Primary Health Network (the "PHN"), The George Institute for Global Health and Improvement Foundation Australia, funded through a Health Research Award from Bupa Health Foundation. In Australia, Primary Health Networks (PHNs) are meso-level (supporting) organisations contracted by the Australian Government to improve access to primary care services for regional patients and coordinate with local hospitals to improve the overall operational efficiency of primary care.

The overall objective of the QPulse project was to enhance the implementation of CVD risk management guidelines in general practices. The project faced significant implementation challenges, and quantitative analysis of the quality improvement outcomes failed to demonstrate any changes in risk factor documentation, risk factor prevalence, attainment of physiological targets or prescribing for risk reduction after the intervention [23, 24]. However, a detailed sub-analysis of the data did demonstrate significant variation between practices. Some high performing practices showed selected areas of improvement in patient data collection, such as recording weight and height [23]. Gaining a knowledge about individual general practice enablers and barriers is key to developing improved QI strategies for Australian general practices. This qualitative study aimed to explore the specific implementation barriers and enablers encountered by participants in the QPulse project. The overall goal was to inform future health policy and funding initiatives for QI in the General Practice setting.

## Methods

### Study context

The QPulse QIC project (Central and Eastern Sydney General Practice Quality Improvement Network: building a sustainable model of QI to achieve reduced cardiovascular disease in the primary care setting) was conducted in 2015–2018 [23, 24]. This mixed-methods research project was performed in accordance with the Declaration of Helsinki and was approved by the University of Notre Dame Australia Human Research Ethics Committee (UNDA HREC) (reference 014105S). Signed agreements with participating practices and interviewees were obtained, and the committee granted a waiver for patient-level consent. The study was registered with the Australian and New Zealand Clinical Trials Registry, ACTRN12615000108516, UTN U1111-1163–7995.

The current qualitative study was conducted following the completion of the QIC component of the QPulse project. Detailed information about QPulse QIC recruitment, interviewees and data collection have been reported elsewhere [23, 24].

In brief, QPulse was a series of three QIC's, each lasting six months. The QICs were designed to be overseen and delivered by the PHN into general practices using an abbreviated version of the typical 18-month duration QIC [15, 18]: comprising educational workshop followed by six monthly data audit reports alongside practice generated Plan, Study, Do, Act (PDSA) cycles [25]. The QPulse QIC's used existing medical audit and decision support software [26] to assist participating General Practices in measuring and managing CVD risk factors. A timeline and Figures describing the recruitment and project rollout of the QPulse project can be seen in Figs. 1–3, Supplementary file 1.

### Qualitative interviews

After the rollout of the QPulse QIC, nineteen semi-structured interviews were conducted using a purposive selection of participants involved in its implementation, including practice managers (PMs), nurses and GPs from the participating general practices, and program officers, IT support personnel and managers at the PHN. Purposive sampling was used to ensure the study achieved broad representation from participants across the full range of both general practices and the supporting organisation. Twenty-two people were approached by the first author by phone or email, inviting them to participate in a 30-min interview, face to face or via the phone (as preferred by the interviewee). Three invited GPs opted not to participate due to lack of time. All nineteen interviewees were emailed a set of questions (see Tables 5 and 6 in Supplementary file 2) to assist them in preparing for the interview on enablers and barriers to participating in the QPulse project. One PHN interviewee asked to respond to the questions in written format in preference to verbal responses. The oral interview was semi-structured, and interviewees were invited to elaborate on any question that they felt would be helpful to explore further. Each interview was audio-recorded and then transcribed using a transcription service. Transcriptions were shared with the interviewees to ensure they were seen as accurate and they were agreeable to the contents being used for the study.

Two GP interviewees were interviewed together at their request, but all other interviews were conducted as one-on-one sessions.

### Data analysis

Our analysis drew on the Kraft et al. Complex Systems Improvement (CSI) framework [27]. This framework was selected for its relevance to the context and complexities of the Australian general practice environment. The framework identifies four health system levels that align with the successful implementation of change—environment, meso-level organisations, microsystems, and patients and their caregivers [27]. The analysis in this study primarily concentrated on three levels in the CSI framework, i.e. the environment, the meso-level organisation (PHN) and the microsystem (general practice).

The CSI framework also identifies five domains for evaluating a change-making intervention in the health system. These domains include strategy, culture, structure, workforce and technology. For the purpose of this study: "Strategy" addresses alignment of the improvement intervention with the strategic intention of interviewees. "Culture" looks at the norms, values and beliefs of interviewees. "System" addresses infrastructure in place to enable interviewees to learn new practices, spread best practices, and continuously measure performance and improve processes. "Workforce" looks at how people, tasks, tools and technologies, organisational conditions, and physical environment affect the adoption of the intervention. "Technology" specifically addresses the role that IT and electronic medical records play in the adoption of new processes. We applied the framework to examine the change intervention experience rather than describe the implementation sequence.

Four researchers independently read and analysed the interview transcripts; this comprised the principal investigator of the QPulse project (first author) and three researchers who had not participated in the design or implementation of QPulse; one is a co-author of this paper, and two are noted in acknowledgments. All were approved to contribute to the analysis via the UNDA HREC process. Each researcher manually coded interviews to develop core themes and observed patterns in the data. Two co-authors (CH and EB) reviewed the identified themes and systematically analysed them against the Kraft et al. CSI framework [27].

After analysing the transcripts from all 19 interviews, the research team was confident data saturation had been achieved, with no need for further interviews.

## Results

Nineteen participants were interviewed after providing the research team with written and verbal consent to participate in the qualitative study. Individual and practice demographics for each interviewee are provided in Tables 1 and 2.

**Table 1:** Interviewee characteristics of interviewees from the Primary Health Network and general practices

Interview interviewees from PHN (*n* = 7)	
Female	4
Project Officer	2
Team Manager	2
Executive Officer	1
IT Support Officer	2
Interview interviewees from general practices (*n* = 12)	
Female	9
Practice nurse	1
Practice manager	1
General Practitioner	10
Practice size (number of regular patients)	
< 2000	1
2001- 4000	1
4001–6000	3
6001–8000	3
8001–10,000	2
10,001–20,000	1
> 20,001	1
Previous QI experience	6

**Table 2:** Practice demographics of general practice interviewees

Wave	Gender + Role	Practice size **VS**mall **S**mall **M**oderate **L**arge	# downloads	Billing	# GP’s In practice	Practice Nurse	Allied Health On site	PM / Admin support	Prior QI
1	F GP	M	16	Mixed billing	6	1 × PN	Y	PM + Admin support	Y
1	F GP	L	16	Bulk Billing Corporate	7	2 × PN	Y	Corporate PM + Admin support	N
1	F GP	VS	16	Mixed Billing	1	1	N	PM + Admin support	N
1	M GP	M	17	Bulk billing Corporate	2	0.5 × PN	Y	PM + Admin support	N
1	F GP	S	18	Bulk billing	2	No	Y	No PM + Admin support	N
1	F GP	M	24	Mixed billing	16	2 × PN	Y	PM + Admin support	Y
1	M GP	S	14	Mixed billing	4	1 × PN	Y	PM + Admin support	Y
1	M GP	M	15	Mixed billing	5 GP	1 × PN	N	PM + Admin support	N
1	F GP	S	14	Mixed billing	4 GP	1 × PN	N	PM + Admin support	Y
2	F GP	S	13	Mixed billing	1.5 GP	1 × PN	N	PM + Admin support	Y
2	F PN	S	13	Mixed billing	1.5 GP	1 × PN	N	PM + Admin support	Y
1	F PM	M	25	Mixed billing	16 GP	1 × PN	Y	PM + Admin support	Y

### Qualitative data analysis: complex system improvement framework

The CSI framework was used to identify insights and issues that affected the QPulse intervention environment, and the experience of implementation for the general practices and PHN (health system levels) examined across the five domains.

A summary of the key findings of our analysis aligned to the Complex System Improvement Framework is presented in Table 3. Incentives were identified as a key enabler across all five domains and these findings are summarised in Table 4.

**Table 3:** Study findings analysed within the Framework for Complex System Improvement proposed by Kraft, Carayon [27]

	**Goals and strategies** (incentives, priorities, opportunities for change	**Culture** (values, beliefs, norms)	**Structure of learning** (infrastructure to support continuous learning and improvement)	**People, workflow and care processes** (role optimisation, processes of care, standard workflows)	**Technology** (information services, electronic health records)
**Patients and caregivers**	Support GPs in improved CVD prevention and care Engage GPs in Quality Improvement data collection and scrutiny	Highly variable, a key determinant of success Enrolled GPs personally motivated to improve their practice	GPs have ongoing structured CPD with emphasis on evidence-based care, support from the college GPs supported by PHN staff during implementation	Increased workload for GP practices would have appreciated more support, e.g. from PHN	Healthtracker, Topbar often needed troubleshooting (PHN generally prompt with this) Practice members sometimes experienced problems due to knowledge deficits More incentive required to encourage the sustained use of the tools by GP or PN
	Patients voice was not captured in this study: no ability to record their role in adopting preventive care strategy	Patients values and beliefs were not measured in this study – they were seen as recipients of their GP’s advice to be educated in preventive health by their GP	GPs stated Healthtracker to be educational and engaging for patients, but this was reported from the perspective of the GP and PN	Patient-centred workflow processes lacking and should be included in the next stage of the design	Healthtracker was noted to be engaging and valuable for patient use during consultations
**Microsystems (small units where care is delivered)** **i.e. Practice level** **(The General Practices)**	GP practices vary widely in nature (size, internal supports, team culture/lack thereof, business models etc.): opportunities for change are affected by this on an individual level. Solo and large practices are seen to struggle more with the adoption of systematised QI practices	GP practice culture and leadership key to implementation The culture was noted to be very variable Level of engaged leadership variable Practice culture/ circumstances dictate or limit possibilities for change in systems. Individual GP priorities appeared to override the ability to introduce changes in practice and systems	Practices required hands-on support – and would have appreciated more proactive help from PHN staff (e.g. regularly scheduled visits, facilitated networking, more in-practice teaching about QI and clinical topics requiring improvement, structured learning using practice data)	Some practices were agile concerning role optimisation and adoption of new processes Successful implementation required effectively engaging PNs and PMs as well as the GP. Change leadership by a GP ± PN or PM was key to success	Software used varied between practices, sometimes incompatible They were seen as time-consuming It quickly became a barrier due to the time required PHN was generally competent in resolving practice-level IT problems but was often left out of the loop
	Individualised approach required				
**Meso-level Organisations (supporting microsystems)** **i.e. PHN, RACGP**	Clear guidelines, readily accessible, need for improvement universally agreed	The identity and nature of PHN were in flux at the time of the study. The need for established and trusted relationships between practice and PHN was identified as key to ongoing success	Seen as the role of the PHN by practices PHN did not visualise its role consistently throughout this project due to a lack of prioritisation and resourcing by senior management for this work	Strategic leadership by executives aligned to QI was fundamental Personnel selection and support at PHN may have been non-optimal	IT support by PHN key to implementation – PHN offered excellent IT support in most cases, but GP’s did not always utilise this service
**Environment** **(policy, payment, regulation)**	Clear guidance from the Department of health to prioritise this work and part of the new PHN contract. Minimal reimbursement available to assist practices or PHN to fund the work adequately	“Quality Improvement” is part of Australian Primary health care policy documents but not incentivised for individual GP’s nor adequately funded within the entire primary care health system	Adversely affected through changes in ML to PHN They are not funded. GPs have to do mandatory CPD to maintain Australian Medical registration. RACGP has mandated 1 QI activity every three years for each GP to maintain specialty status and registration	QI Practice Incentive Payment is available for accredited General Practices but not yet linked to any tangible programs related to improvements in services	No current funding is available for practices to support the adoption of any specific technology PHN contracted to provide generic “QI support” to general practice by the Federal Health department but no actual funding stream to implement

**Table 4:** Quality improvement incentive framework

	**Microsystems** - General Practices	**Meso-level organisations** - PHN	**Environment** - Government Policy and funding (State and National) - Professional
**Goals and strategies**	Funding incentives to support targeted Quality Improvement projects within general practice that include Preventive care	Clear guidelines from the Department of Health regarding support for QI project Provide funding in contracts to deliver these strategies, Target Improvement projects with universally agreed goals as well as locally identified projects	Align Health Policy Strategies with funding into primary care Reform Primary care funding policy
**Culture**	Funding incentives to create Quality Improvement “cultural” buy-in for **all** styles of General Practices Participatory – enable adequate resourcing ◦ Practice Administration ◦ Individual Practitioner	Enable provision of flexible support for general practices to assist in the establishment of systematised QI in all general practices – solo/group/corporate	Align Health Policy Strategies with funding into primary care Reform Primary care funding policy
**Structure of Learning**	Professional Incentives for attendance and active participation in practice-based QI CPD activities - Professional points / Registration - Financially linked to income/practice payments - Access to clinical / IT services	Contracted to provide individualised support to the general practices to systematise QI education programs and clinical pathways and processes	Professional bodies to set Quality Improvement benchmarks and KPI as part of the registration and accreditation requirements
**People, workflow and processes**	Funding Incentives to provide Team-based solutions for systems of patient care	Provision of QI CPD - Ensure CPD programs link directly with QI programs and overall QI systems change Provide financial incentives to Practices who participate in QI programs/data collection Provide reminders and support for data reports and PDSA related systems change	Reform Primary care funding
**Technology**	Funding Incentives to encourage practices to adopt technology to assist with QI programs	Provide IT support for new technology	Practice incentive payments to align with Technology requirements

### Goals and strategies (incentives, priorities, opportunities for change) for improved adoption of CVD risk prevention guidelines

All interviewees in the study aligned with the QPulse goal of decreasing CVD related mortality and morbidity, and saw this as a priority for QI work in their community. However, they reported difficulty in adopting QI process into regular work systems due to lack of any other tangible incentive.

It was reported that GP interviewees signed up for the QPulse study because they were personally interested in improved preventive care and individual patient health outcomes.

*"It was an opportunity to become more proactive rather than reactive, … it's too much reactive care in general practice, I think, even though we're obviously aiming to be preventative, often in the day to day running of a practice, they don't happen." GP9*

Still, doing this work as part of usual business proved difficult for most. The lack of financial incentives meant that it was ultimately not given sufficient priority by the GP practice staff or the PHN. In particular, QI was seen as time-consuming and low priority to systematise into existing business models.

*"QI projects currently happen outside of consulting and in general practice the only way that you can have money coming in is to be seeing patients and providing services…..I think funding incentives for QI projects would be good because then you can then allocate some time." GP6*

### Culture (values, beliefs, norms)

The overriding organisational QI culture was reported as key to implementation of QI activities for both the general practice and the PHN. QI culture being defined in this context as an environment where the organisational team hold a shared understanding and belief in the value of doing QI activities designed to evaluate and/or improve healthcare delivery and outcomes [28].

There were significant cultural differences noted between the participating practices. While initial interest in and enrolment into the project was driven mainly by an individual GP or Practice Manager,having a practice culture which aligned with a supporting QI activities was reported as an essential factor for successful implementation of QI, rather than the size or location of the practice. One GP interviewee described a practice culture characterised by clearly defined leadership, collaboration with all the staff (primarily via regular meetings and discussion around identified areas of improvement) and commitment to try new initiatives.

*"It really comes down to the culture within the practice, who is the real leader, who is the driver in the practice… with QI, for it to be really successful, you need all of practice engagement, but you really need to have somebody who is going to take the reins on that." (PHN1)*

The general practice interviewees highlighted that the most critical determinant for whether or not they could implement and sustain the QI work was the culture created by their significant leaders. Identified influential leaders were usually a GP (owner or designated "lead") but also noted to be the Practice Manager or Practice Nurse.

Interviewees reporting a pre-existing QI culture also noted increased practice engagement during this project. Practices with no prior experience of QI reported difficulty engaging GPs in the QI process. In particular, corporate^2^ style practices did not appear to have systems to enable the adoption of QI to improve patient outcomes by contracted GP's. This style of practice was also noted to lack a practice culture designed to engage the entire team with each of the identified changes to achieve an improvement. On the other hand, practice teams who had previously embraced QI were more enthusiastic about being involved. They utilised established clinical audit and review systems to identify what needed to be done, by whom and how to check whether it achieved the desired outcome. Some interviewees reported recruiting staff aligned with QI culture and had a policy of ensuring the entire team received regular updates about QI projects. Conversely, in practices that described a lack of commitment to defined leadership or QI, project uptake was less enthusiastic and difficult to disseminate to the GPs working in the practice. One interviewee from a larger corporate style practice who was personally motivated by an interest in CVD noted that implementing practice-wide change was only possible with the co-operation of the owner, practice manager, nurse and secretaries. They reported that this had not been evident in their practice during QPulse. They noted that it was challenging to engage the GPs to do anything that might involve extra work. This corporate style of practice enabled GPs to work as individuals with no culture around overriding clinical governance or accountability around the quality of care delivered to their patients.

Several interviewees who discussed the clinical benefits of working within a group of GPs aligned with QI contrasted with a solo GP who noted interactions in her team tended to revolve around practice management rather than clinical issues. Peer support for QI in clinical management for the solo GP was gained through external activities such as PHN organised professional development. This practice reported difficulty in achieving sustainable implementation of QI processes, despite having authority and clinical leadership in adopting change. The constant demands upon the GPs' time from acute issues precluded what were perceived as optional, less essential activities.

At its most pragmatic, a lack of consensus or accountability regarding clinical input from peers meant that introducing QI was seen as too time-consuming from the clinician viewpoint – particularly given the lack of visible or measurable benefits over the longer term.

*"Unless I can see an immediate necessity for it, I'd rather not do it…. (GP3)"*

A PHN interviewee also noted that their meso-level organisation needed to have a cultural shift from seeing QI as an optional add-on and instead identifying it as a core process that integrates into all projects, alongside building relationships with the general practices in their footprint.

*"QI should be embedded in everything we do…" (PHN4)*

### Structure of learning (infrastructure to support continuous learning and improvement)

Overall, most GP interviewees did not report having a structured approach to continuous learning and quality improvement within practices. Many GP interviewees described a lack of clinical leadership within their Practice team, operating as a group of siloed independent GPs with no structured approach to education or support by their employer. Most Practices held some face-to-face meetings as an entire Practice; however, the purpose and intention of the sessions varied from practice to practice depending on the owner's preference. Corporate practice interviewees noted regular lunchtime meetings sponsored by Pharma with no relationship to their individual or collective learning needs. Several interviewees said they would have appreciated short, practice-level presentations from the PHN, particularly after the QI workshop, to assist with how to implement what had been presented.

However, some interviewees noted the difficulty in getting GPs together to meet as this was unpaid time and so not seen as a priority for contracted GP's. Specifically, there was no time available during practice hours for scheduling meetings around QI topics. PHN interviewees also noted the difficulty in gaining access to general practices to talk to GPs – they reported being heavily reliant on communication via the non-GP staff such as the practice managers and nurses. Education was seen as an individual responsibility for the GP's rather than as part of the Practice responsibility.

The PHN interviewees also noted the lack of resources to provide educational support, despite acknowledgement by the PHN senior executive that the provision of face-to-face support was key to engagement and implementation of programs with GP's and practices.

*"Support from an individual at the PHN was essential and a main driver of the project" (PM1)*

Another barrier noted by interviewees to the adequate provision of PHN services to practices was the regular turnover of key project staff. This led to the need to retrain and upskill new project staff, loss of corporate memory, and inadequate capacity to fully undertake the required scope of GP support programs. In most cases, the priorities of each general practice were reported as influenced by the lead GP, but with implementation usually handed over to PM or PN. All interviewees felt that a lack of tailored practice support hampered the implementation of the QPulse project activities. Positive adoption of QI and change in systems were reported as more likely where key practice staff had an inherent interest and capabilities in clinical data management and computer software skills.

While interviewees reported initially completing the PDSAs [29] as requested, these were reported as negative experiences. The PDSAs were described as tedious, time-consuming or repetitive—with no one adopting this methodology as a systematised way to assist in QI activity, despite acknowledging their value in targeting change. The PHN interviewees also reported very little engagement with the PDSA process.

*"Getting practices to submit PDSAs was very difficult …I think that GPs think it is too time-consuming…If we can come up with a less time-consuming version, I think they would be more willing to complete it." (PHN3).*

Some interviewees did note positive changes within their practice following the implementation of previous structured QI programs, including increased coding of diagnosis and the ability to track improvements over time with reports that included all of their data and charted improvements. The opportunity to engage with the data was limited, with only intermittent reporting amongst the participating practices due to the IT and scheduling problems associated with the software. PHN interviewees also noted that data extractions without the follow-up provision of monthly reports and targeted education provided little long term value for the practices.

When asked about attending education, training and networking sessions designed to upskill general practice staff to do QI work, most GP's reported that they favoured face-to-face engagement. However, this was also reported as a significant barrier to participation as there were never mutually convenient times or places for everyone to attend. For QPulse, this was reflected in the poor attendance rates by participating practices at scheduled training and support sessions despite prior agreement to attend.

*"The CPD workshops were good at engaging members but it was very hard to get them there" (PHN4)*

### People, workflow and care processes (role optimisation, processes of care, standard workflows)

Although it was confirmed in the interviews that all interviewees had engaged with baseline requirements of the QPulse project (measurement of baseline data, initial goal setting, setting up (at least one) PDSA cycle and then reviewing goals). It was also evident that only two practices had implemented practice workflows to achieve a sustainable QI process. Most GP interviewees reported that they saw it as just another extra thing to do, rather than an opportunity to improve their data or health outcomes.

*"QPulse was lighter touch than we would have liked, like this was supposed to be much more engaging program than what it ended up being." (PHN4).*

Two interviewees from the most engaged practices also discussed the difficulty of achieving sustainable QI. They cited both lack of tangible incentives (for practice management and GP employees) and dedicated time to do this work. PHN interviewees identified the need to provide long-term assistance in this work rather than brief interventions rolled out with no system or solutions to achieve sustainability.

They noted that most individual GPs are not interested in practice management and workflow systems and instead are focused on getting through their daily acute clinical care workload. The need to align appropriate resourcing by the PHN to enable role optimisation for the frontline PHN project officers was highlighted as key to the implementation of QI by PHN and GP interviewees. All interviewees noted the lack of resources allocated to QI work by the PHN.

Lack of clarity around the roles and responsibilities of PHN staff was highlighted by PHN interviewees as another barrier to QI implementation. One interviewee observed that a lack of clear guidance by team leaders about the QPulse project had resulted in a lack of motivation and uncertainty in terms of what each staff member should be setting out to achieve and the outcomes they were accountable for delivering.

*"QPulse became a mini-project, carried out by a lone project officer, separated from the "core business" of the PHN" (PHN5)*

PHN interviewees identified specific enablers included strategic use of flexible funding streams (to fund QI work). Key barriers were the high staff turnover, lack of engagement and skills in QI work by crucial staff (particularly frontline project officers), full time versus part-time roles (continuity of functions) and staff managing competing priorities with minimal time allocation to assisting with "add on" QI projects.

In addition, it was noted that at the start of QPulse, three meso-level GP organisations (formerly known as Medicare Locals) were merged to form one PHN increasing the number of practices that fell within the remit of individual PHN project officers. This appeared to exacerbate their difficulty in meeting project and practice expectations. For QPulse, one project officer was responsible for overseeing 40 practices in a role funded at three days per week.

PHN interviewees also noted that QI support needed to be better tailored to individual practice needs and priorities rather than directed by the preferences of specific PHN projects.

*"Lack of funding for the PHN to adequately resource QPulse together with lack of financial incentives for practices to engage was seen as the major barrier to getting things happening" (PHN2).*

The PHN interviewees discussed the importance of prioritising engagement with people in the practice who are responsible for the oversight of systems of care.

Several mentioned that a provision of more regular updates and visits from the PHN might have helped maintain the prominence of this work amongst all the other competing priorities of the busy GP practice.

*"without the reminders from the PHN…it doesn't happen". (GP1)*

Significantly, GP interviewees noted the additional workload arising from QI was not sustainable in the long term without some tangible incentive for interviewees – both for the individual GP and the practice team. Incentives might be both financial and aligned with accreditation and registration. The particular challenges of sustained engagement when the practice operated as a group of independent contractors was also noted, especially with a lack of obvious financial incentives.

### Technology (information services, electronic health records)

The use of technology tools to aid QI, such as Healthtracker, the clinical decision support tool, was reported as crucial in successful implementation but was also a cause of failure and disengagement, often needing additional time investment in troubleshooting. There were varying levels of IT ability and IT difficulties experienced within the GP practices and by PHN staff. Barriers ranged from poor IT connectivity, incomplete data entry, challenges with using the software tools, and achieving sustained usage, specifically for QPulse, adopting the new technology (Healthtracker) during clinical patient encounters. From a practice perspective, most interviewees saw the PHN as an essential resource, particularly concerning the installation and troubleshooting of the Healthtracker software.

The importance of good relationships with the PHN was made clear by several interviewees, both as a supportive IT support resource (e.g. installation of PenCAT and troubleshooting problems with Healthtracker) and as a source of reminders to do the monthly data extractions and data review. GPs appreciated the assistance provided by the PHN at the point of software installation, noting that this ensured the program was useable by the general practice participants.

*"Healthtracker needed GPs sure it.. wasn't a white elephant…that no one could use" (GP2).*

Many GP interviewees stated that learning to use new technology was a barrier, yet also noted the decision support tool, Healthtracker, was user friendly and appealing to both GP's and patients. However, Healthtracker did not always run as intended in some practices, with several interviewees reporting that they had experienced problems, although these were usually readily solved by the PHN contacts. It was evident that none of the GP interviewees achieved sustainable adoption of Healthtracker despite acknowledging its value-add during consultations.

Software incompatibility was also cited as a significant barrier, with no on-call IT support to troubleshoot a solution. Ongoing and often unresolved difficulties encountered included software crashing with updates, lack of automation with data extractions and reminders, inability to access or use the PenCAT tools, and problems setting up and training all practice team members.

Some interviewees also noted that access to the PenCAT Data extraction tool could be difficult. It was only available on one computer terminal within a practice providing a barrier for easy implementation of the QI process.

One GP interviewee expressed her disappointment when there were problems with data extractions and exports, resulting in a disruption in ongoing data reports.

*"we put all those figures in for 12 months…I thought we'd be reviewing all our data to see if we were better but they stopped our access…." (GP4)*

Most GP interviewees found that regularly submitting data to the PHN was beneficial for setting up a pattern of QI work.

*"certainly having that done is very important to see how we're going "(GP2)*

Still, they found the ongoing time requirements challenging without any financial incentive to compensate for this task's administrative burden in the too-hard basket.

The QPulse project did not examine patient barriers to medication utilisation nor the adoption of recommended lifestyle measures as these data fields were not extractable from the GP medical records.

*"patients were very keen to be involved – but they wouldn't realise the risk and TopBar (Healthtracker) was a great way of visually explaining this to them" (PN1)*

However, GP interviewees discussed improved conversations with patients when using the Healthtracker point of care tool, which they stated achieved better engagement in discussions regarding preventive care strategies.

## Discussion

The QPulse interviews provided an opportunity to understand the why and what happened of the project's failure to quantitatively demonstrate changes in the measured data outcomes as previously reported [23].

What is the daily experience of real-world general practice that prevents the adoption of routine CVD preventive care? QPulse interviewees understood improving CVD preventive care provided them with an opportunity to decrease mortality rates linked to CVD in Australia. Yet, they were unable to demonstrate any tangible change in the recorded risk measures or prescription of CVD preventive care [23]. A 2021 AIHW report looking at data from > 5700 Australian general practices showed disappointingly similar results, with only 48.5% of patient records showing cardiac risk measures [14]. This figure has not shifted over the last 15 years [8, 24, 30] despite the introduction in 2019 of a Government-funded Heart Health check by GPs [31]. This study, together with the AIHW data demonstrate a need to change health policy strategic approaches, such as implementing incentives alongside quality improvement projects to address barriers revealed by this analysis.

There are differences between the implementation of change exemplified by the original Kraft et al. article and those found in this study. QPulse relied on general practices opting into the project, whereas Kraft et al. focused on a mandatory, all-practices, system-wide implementation. The critical role of the supporting organisation in the QPulse project was complicated by the PHN being a new and evolving entity rather than an established organisation with a clear strategy for supporting and implementing QI projects.

Both general practice and PHN's are reliant on their external environment to provide the incentive required to enable work outside of the current fee-for-service model of primary healthcare.

Our analysis identified incentives as critical enablers across all five domains for improvement strategies in both general practice (microsystem) and the PHN (meso-level organisation),which included both dedicated funding for QI directly into general practice, such as the financial incentives introduced into Sweden in 2016 [32] and mandatory continuing professional development that incorporates quality improvement, to encourage changing clinical practice aimed at improving patient outcomes [33].

The analysis also identified a range of themes across the five domains of the CSI framework that align with current national and international research on implementing quality care initiatives within primary care settings [34–38]. The themes identified from this study include the crucial need for leadership, both at the practice and PHN, and the provision of tailored education and support for each practice setting. The need for better communication systems and trust amongst all staff and project officers is essential, including the need to address many GP's' lack of readiness for change.

Implementing change also requires a paradigm shift from individual practitioner care toward team-based care alongside a longer-term commitment to achieving sustainability rather than rolling out a series of independent projects, as found in other healthcare settings [38, 39]. Other resonant themes were the need for better IT systems and support, such as integrated electronic health records, decision support tools and data reports, and funding models designed to support sustainable changes in general practice systems [38–40]. In QPulse, the culture of each general practice was crucial to implementing the QI program. Each practice had distinct and unique characteristics affected by previous QI experience, practice ownership and their underlying philosophy regarding patient-centred, team-based models of care versus physician autonomy. It was also evident that even the QI culturally engaged practices needed financial support to normalise QI work to sustain a constantly growing portfolio of QI projects. There is evidence of the flow-on effect of the fee-for-service funding model, which provides a perverse disincentive for most GP's to participate in non-face-to-face care, such as QI activity [40]. This will need to be addressed, via funding reformation, if implementation and sustainability of QI programs are to be improved.

Overall implementation of the QPulse project was adversely affected by the timing of its rollout. Specifically, the initial rollout coincided with a significant change in contract, funding and structure of the meso-level organisation. As a result, QPulse was sidelined into being a siloed QI project rather than becoming part of a strategic QI program for both the general practices and PHN staff. This affected the implementation for all participants in the study. The current study also had specific limitations in that it was conducted in one urban PHN, with a limited number of general practices located in this footprint and thus cannot reflect barriers specific to rural or regional areas.

## Conclusions

A strategic, evidence-based approach should be taken for future funding of primary care QI programs. The need for incentives prioritising the adoption of QI, such as funding for both infrastructure and time, has been identified as crucial in the current Australian healthcare system.

Implementation strategies should flexibly address and support the required incentives to address the identified range of issues specific to general practice setting: culture and readiness for change, practice-based education programs, leadership training and accessible IT support. PHNs need to be contracted to deliver these programs. Staff can only do this if the organisation has contractual obligations and funding to enable this level of support proactively.

## Supplementary Information

**Additional file 1: Figure 1. **QPulse timeline. **Figure 2.** QPulse Practice Recruitment.** Figure 3.** Flowchart QPulse QIC intervention.

**Additional file 2: Table 4.** Interview questions for stakeholders in general practices.** Table 5.** Interview questions for stakeholders in Primary Health Network.

## Data Availability

The datasets generated and/or analysed during the current study are not publicly available but data may be made available to interested parties from the corresponding author on reasonable request.

## Abbreviations

CSI: Complex Systems Improvement framework
CVD: Cardiovascular disease
GP: General practitioner
IT: Information Technology
PDSA: Plan-Do-Study-Act
PHN: Primary Health Network
QI: Quality improvement
QIC: Quality improvement collaboration
UNDA HREC: University of Notre Dame Australia Human Research Ethics Committee

## Footnotes

1 Guidelines-recommended treatment was defined as: among high-risk patients, prescription of a BP-lowering medication and a statin, and among patients with established CVD, prescription of a BP-lowering medication, a statin and either an antiplatelet or an anticoagulant.

2 Corporate style practice is used here to refer to those General Practices owned by an incorporated entity rather than owned by one or more of the General Practitioners working in that same practice.
