# Supplementary material for: Learning from the implementation of a quality improvement intervention in Australian general practice: a qualitative analysis of participants views of a CVD preventive care project
Source: BMC Prim Care. 2022 Apr 14;23:79. doi: 10.1186/s12875-022-01692-0 (PMC9011978; doi:10.1186/s12875-022-01692-0)
Supplement: Supplementary file 1 — Additional file 1: Figure 1. QPulse timeline. Figure 2. QPulse Practice Recruitment. Figure 3. Flowchart QPulse QIC intervention. [file 12875_2022_1692_MOESM1_ESM.docx]

**Supplementary Figures (1,2,3): Figure 1 QPulse timeline**

**Figure 2: QPulse Practice Recruitment**


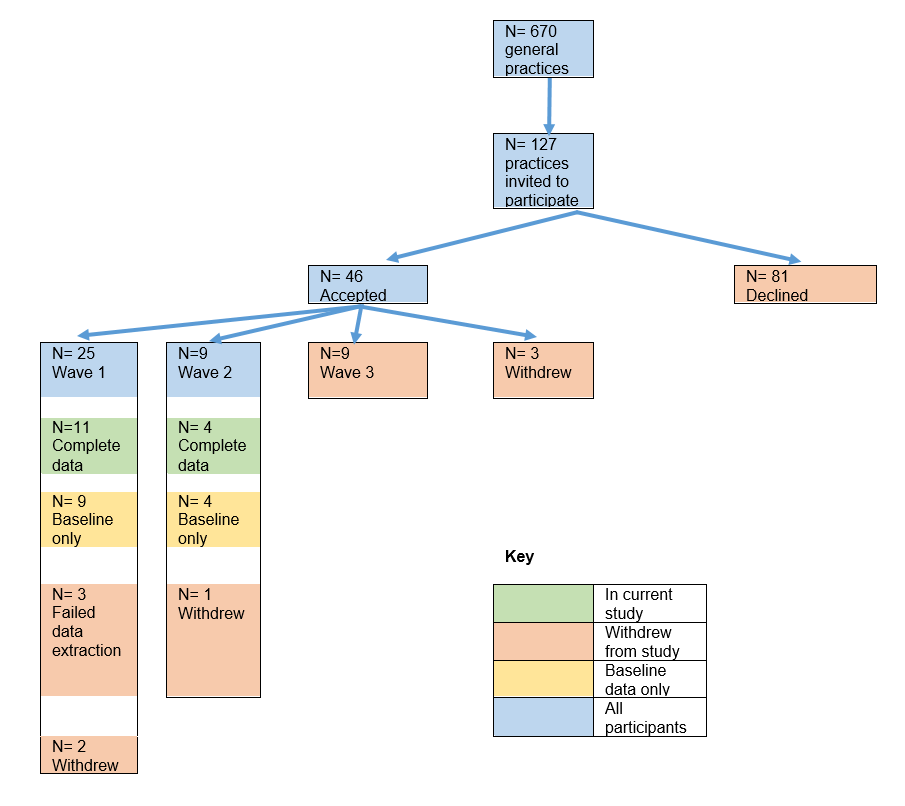


**Figure 3: Flowchart QPulse QIC intervention**

46 General Practices expressed interest in QPulse

34 General Practices enrolled and participated in QPulse QIC program

(25 Wave 1 + 9 Wave 2)

Following the workshop, each General practice asked to submit

- monthly clinical data extractions x 6

- monthly submission of a PDSA

Project ends for each practice 6 months after first workshop

General practices invited to participate in monthly webinars to share PDSA’s, assist with QI and establish a network of support

GP teams asked to each send 2 attendees to 3 x 2 hour QI workshop (time “6”)

Each General practice submitted baseline data 6 months prior to workshop

Practices provided with:

IT Support

PenCAT data extraction tool

Health Tracker

PHN provides

support for QI work at practice level

QI education and CPD at workshops

Monthly data reports

Webinars ceased after 3rd month due to lack of engagement by practice teams.

Final Quantitative data received -

28 x baseline datasets

15 x pre and post datasets

GP teams invited to participate in interviews about the process of QI participation after completing the QIC timeline

(12 interviews conducted)

PHN staff invited to participate in interviews about the process of QI in the Primary care environment

(7 PHN interviews conducted)
