# Supplementary material for: Learning from the implementation of a quality improvement intervention in Australian general practice: a qualitative analysis of participants views of a CVD preventive care project
Source: BMC Prim Care. 2022 Apr 14;23:79. doi: 10.1186/s12875-022-01692-0 (PMC9011978; doi:10.1186/s12875-022-01692-0)
Supplement: Supplementary file 2 — Additional file 2: Table 4. Interview questions for stakeholders in general practices. Table 5. Interview questions for stakeholders in Primary Health Network. [file 12875_2022_1692_MOESM2_ESM.docx]

**Supplementary Tables (4, 5):**

**Interview questions for stakeholders from general practices and Primary Health Network**

**Table 4 Interview questions for stakeholders in general practices**

Why did their practice enrol?

What happened in their practice during the project? What went well and what was difficult

What members of the practice engaged with the project / tasks – who and why they were involved.

Who set/s the priorities for doing tasks in the practice

How did the practice work together as a team (or not) on the project?

What could have been improved to assist them in participating in the project?

Were they continuing to do QI work?

Try to provide some details around the following

Barriers and enablers to the implementation

What worked well and what did not work well?

What would they want to do differently if involved in a similar project in the future?

What would they want done differently to assist in their participation in the future?

Leadership in their practice

Organisational QI Culture

Funding Incentives to do this work

Data- access and ability to use – are they doing anything now?

Clinical Systems – have they done any work on this area in their practice?

External support – how has the PHN or any other organisation assisted them in any way to do this work?

**Table 5 Interview questions for stakeholders in Primary Health Network**

Outline their role at the PHN for Q Pulse (or similar QI projects)

What was their experience of the project implementation – what worked and what was challenging

What was their experience of QI project implementation more generally – what worked and what was challenging

Then ask some more specific questions about the following areas:

Barriers and enablers to the implementation

What worked well and what did not work well?

What would they want to do differently if involved in a similar project in the future?

What would they want done differently to assist in their participation in the future?

Leadership in the workplace

Organisational QI Culture

Incentives to do this work from Executive level / funding body

Data- access and ability to use – are they doing anything now?

Clinical Systems – have they done any work on this area in the PHN?

External support – has any other organisation assisted the PHN in any way to do this work?
